# The Presence and Extension of Myocardial Fibrosis in the Undetermined Form of Chagas’ Disease: A Study Using Magnetic Resonance

**DOI:** 10.5935/abc.20180016

**Published:** 2018-02

**Authors:** Marcia Maria Noya-Rabelo, Carolina The Macedo, Ticiana Larocca, Admilson Machado, Thais Pacheco, Jorge Torreão, Bruno Solano de Freitas Souza, Milena B. P. Soares, Ricardo Ribeiro-dos-Santos, Luis Claudio Lemos Correia

**Affiliations:** 1 Hospital São Rafael - Fundação Monte Tabor, Salvador, BA - Brazil; 2 Escola Bahiana de Medicina e Saúde Pública, Salvador, BA - Brazil; 3 Centro de Biotecnologia e Terapia Celular, Salvador, BA - Brazil

**Keywords:** Chagas Disease, Chagas Cardiomyopathy, Fibrosis, Magnetic Resonance Imaging

## Abstract

**Background:**

Previous data has shown that patients in the indeterminate form of Chagas disease may present myocardial fibrosis as shown on through magnetic resonance imaging (MRI). However, there is little information available regarding the degree of severity of myocardial fibrosis in these individuals. This variable has the potential to predict the evolution of Chagas’ disease into its cardiac form.

**Objectives:**

To describe the frequency and extent of myocardial fibrosis evaluated using an MRI in patients in the indeterminate form, and to compare it with other forms of the disease.

**Methods:**

Patients were admitted one after another. Their clinical history was collected and they were submitted to laboratory exams and an MRI.

**Results:**

Sixty-one patients with Chagas’ disease, with an average age of 58 ± 9 years old, 17 patients in the indeterminate form, 16 in the cardiac form without left ventricular (LV) dysfunction and 28 in the cardiac form with LV dysfunction were studied. P <0.05 was considered to be statistically significant. Late enhancement was detected in 37 patients (64%). Myocardial fibrosis was identified in 6 individuals in indeterminate form (41%; 95% CI 23-66) in a proportion similar to that observed in cardiac form without LV dysfunction (44%); p = 1.0. Among the individuals with fibrosis, the total area of the affected myocardium was 4.1% (IIQ: 2.1 - 10.7) in the indeterminate form versus 2.3% (IIQ: 1-5) in the cardiac form without LV (p = 0.18). The left ventricular fraction ejection in subjects in the indeterminate form was similar to that of the individuals in the cardiac form without ventricular dysfunction (p = 0.09).

**Conclusion:**

The presence of fibrosis in the indeterminate form of Chagas’ disease has a frequency and extension similar to that of in the cardiac form without dysfunction, suggesting that the former is part of a subclinical disease spectrum, rather than lacking cardiac involvement.

## Introduction

Chagas’ disease is a potentially debilitating endemic problem in Latin American countries and has led to an estimated loss of 750,000 years of productive life.^1-5^ Three stages of Chagas’ disease are recognized: acute, indeterminate and chronic.^4,6^ After the acute phase, about two-thirds of infected patients remain in the indeterminate form, which is characterized by the absence of significant clinical, electrocardiographic or radiological manifestations. However, the disease does not manifest itself in these patients and one-third of them progress to some type of cardiac and/or digestive manifestation, and thus are reclassified as chronic.^7^

Identifying the indeterminate patients that are prone to progress to the chronic form serve as a basis for the research of preventive strategies and a better understanding of the pathological processes that lead to this evolution. However, there are no predictive markers or models capable of estimating the risk of this change.

As such, several researchers consider myocardial fibrosis to be a possible substrate for the development and progression of ventricular dysfunction, arrhythmia, and death.^3,8-10^ The etiopathogenic process that promotes fibrosis involves a multifactorial relationship between the aspects related to the etiologic agent and those related to the host^2,11-14^

The advent of cardiovascular magnetic resonance imaging (CMR), with the use of the late enhancement technique allows for the identification of myocardial fibrosis. Furthermore, it has a gold standard rating with a close anatomopathological correlation.^15^ There is evidence that CMR is able to provide images with high spatial resolution and a high level accuracy in assessing ventricular function.^16^ Previous data have shown that even patients with the indeterminate form may have myocardial fibrosis when tested using CMR.^17^ However, there is little data available on the degree of myocardial fibrosis presented by these individuals, which demonstrates the potential of this variable in the prediction of evolution to the cardiac form. The purpose of this paper is to describe the frequency and extent of myocardial fibrosis evaluated using CMR in patients in the indeterminate form. In order to contextualize this situation, these patients were compared to other individuals in the chronic cardiac form with and without left ventricular dysfunction.

## Methods

### Study population

Individuals with Chagas’ disease were recruited between January of 2012 and December of 2013 at the Chagas’ disease outpatient clinic of the Hospital São Rafael, a tertiary referral center in Salvador, Bahia, Brazil.

Inclusion criteria were: between 18 and 70 years old and a diagnosis of Chagas’ disease confirmed by two positive serological tests (indirect hemagglutination and indirect immunofluorescence). The exclusion criteria were: an acute form of Chagas' disease; previous myocardial infarction, coronary artery disease, or the presence of two risk factors; primary valve disease; terminal renal disease on dialysis; active hepatitis or cirrhosis; hematological, neoplastic or bone diseases and contraindication to magnetic resonance imaging.

The study fulfilled its purpose from the Declaration of Helsinki and was approved by the Ethics Committee of the São Rafael Hospital. All of the individuals signed the Informed Consent Term prior to their participation.

### The Forms of Chagas’ Disease

The indeterminate form was defined by the presence of a *Trypanosoma cruzi* infection in the absence of clinical manifestations. Signs of cardiac involvement were characterized by normal electrocardiograms, chest X-rays and echocardiograms. The cardiac form without ventricular dysfunction was defined by individuals with cardiac involvement known as abnormal electrocardiograms (typically right bundle branch blocks and left anterosuperior hemiblocks) and without left ventricular dysfunction on the echocardiogram. The cardiac form with ventricular dysfunction was composed of individuals with a reduced left ventricular ejection fraction.

### Clinical and laboratory data

All of the individuals underwent biochemical tests, a 12-lead electrocardiogram, a chest X-ray, 24-hour holter monitoring, a cardiac stress test, a Doppler echocardiogram and a CMR. Scores were calculated as follows: functional classes III and IV by the New York Heart Association (NYHA) (5 points), X-ray cardiomegaly (5 points), left ventricular dysfunction, global or segmental echocardiography (3 points), non-sustained ventricular tachycardia during the 24 hour holter monitoring (3 points), low QRS voltage on the electrocardiogram (2 points) and male (2 points). They were then classified into three risk groups according to the score obtained: low risk (0 to 6 points), intermediate risk (7 to 11 points) and high risk (12 to 20 points).^18^

### Cardiac magnetic resonance imaging

A CMR was performed using the Sigma HDx1,5-T system (General Electric; Fairfield, CT, USA). To evaluate how the functioning of the left ventricular, synchronized images were acquired from the electrocardiogram in the expiratory apnea, including in the short axis, long axis and the four chamber planes, in different sequences. The acquisition parameters applied to the dynamic sequence were: a repetition time (RT) of 3.5 ms, an echo time (ET) of 1.5 ms, a flip angle of 60º, a bandwidth of 125 kHz, a field of view of 35 x 35 cm, a matrix of 256 x 148, a temporal resolution (RT) of 35 ms, a cut thickness of 8.0 mm without an interval between cuts. Images from the late enhancement technique were acquired with each heart beat 10 to 20 minutes after the administration of a gadolinium-based contrast agent (0.1 mmol/kg), using a 7.2 ms RT, a 3.1 ms ET, an angle of inclination of 20º, the first phase of cardiac cycle, 16/32 lines per follow-up, a matrix size of 256 x 192, a cut thickness of 8.0 mm, an interval between 2 mm cuts, a field of view of 32 to 38 cm, an inversion time of 150 to 300 ms, a bandwidth of 31,25 kHz, and 2 NEX (number of excitations). The late enhancement technique was used to investigate the presence of myocardial fibrosis, which was estimated by a qualitative (visual) method in accordance with the presence or absence of late enhancement, location and pattern presented; and quantitatively, using percentage values in relation to the total myocardial mass. All analyses were performed with the Siemens Argus program (Simens AG, Munich, Germany).

### Statistical analysis

The categorical data were expressed as numbers (percentages, 95% confidence interval - 95% CI), and continuous data were expressed as mean ± standard deviation (SD) or median and interquartile range (IIQ). Normality was determined by the Kolmogorov-Smirnov test. The comparison of the continuous variables with normal distribution was performed using Student's unpaired t test and Anova (one-way analysis). The Bonferroni method was used for a post-hoc comparison between the groups. Fisher's exact test was used to compare proportions. The Kruskal-Wallis test was used to analyze non-normal continuous variables. Simple linear regression was used in the associations between fibrotic mass and the fraction of the left ventricular ejection. The analyses were performed using the SPSS program, version 20.0 (IBM), and p values below 0.05 (two-tailed test) were considered statistically significant.

As an a priori primary analysis, the frequency and extent of myocardial fibrosis in the indeterminate form was described, and cardiac forms were compared with and without dysfunction. As a post-hoc secondary analysis, the association of fibrosis with the ejection fraction and the Rassi score was tested. Additionally, clinical and laboratory parameters were compared between the indeterminate form and the cardiac form without dysfunction.

## Results

### Clinical characteristics

**Figure 1:**
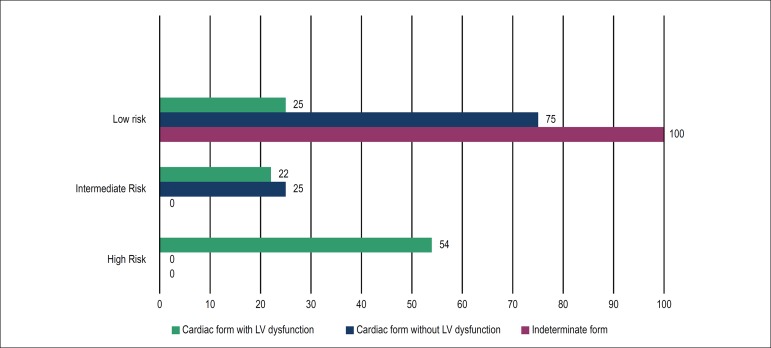
Rassi score in the different clinical forms of Chagas’ disease. LV: left ventricular.

A total of 61 individuals with Chagas' disease, 56% female, with a mean age of 58 ± 9 years old were evaluated. Seventeen of them were in the indeterminate form, sixteen were in the cardiac form without left ventricular (LV) dysfunction and twenty eight were in the cardiac form with LV dysfunction. The majority of the subjects (74%) were in the NYHA functional class I or II, and 4 (6.6%) had concurrent gastrointestinal involvement. 82% were from urban areas, 50 individuals had previously lived in a mud house, and 44 reported contact with triatomine bugs. 64% of the relatives of patients with Chagas' disease reported a testimony. Eight patients used benzonidazole as an etiological treatment. The prevalence of hypertension, diabetes mellitus, dyslipidemia and smoking was similar among the three groups. The median Rassi score was 5 (IIQ: 0 - 11), and was distributed as follows: 36 individuals were classified as low risk (59%), 10 were classified as intermediate risk (16%) and 15 as high risk (25%). Other clinical and demographic characteristics are described in Table 1 and in Figure 1.

**Table 1:** Demographic and clinical characteristics

Variables	Subjects (n = 61)	Indeterminate form (n = 17)	Cardiac form without ventricular dysfunction (n = 16)	Cardiac form with ventricular dysfunction (n = 28)
**Demographic characteristics**				
Age (years), mean ± standard deviation	58 ± 8	59 ± 11	59 ± 9	58 ± 7
Female, n (%)	36 (59)	12 (70)	12 (75)	12 (43)
Lived in a mud house, n (%)	50 (82)	15 (88)	15 (94)	20 (71)
Family members with positive serology, n (%)	39 (64)	11 (65)	14 (88)	14 (50)
Digestive form, n (%)	4 (6.6)	-	2 (12)	2 (7)
Body mass index (kg/m^2^), mean ± standard deviation	25 ± 4	26 ± 4	27 ± 4	26 ± 3
**Comorbidities, n (%)**				
Arterial hypertension	44 (72)	14 (82)	12 (75)	18 (64)
Diabetes mellitus	9 (15)	4 (23)	-	5 (18)
Syncope	6 (7)	1 (6)	1 (6)	2 (7)
Smoking	16 (26)	3 (18)	3 (18)	10 (36)
Congestive heart failure NYHA III/IV	16 (26)	-	-	16 (57)*
**Laboratory characteristics**				
Creatinine (mg/dL)	0.88 (0.7 - 0.99)	0.84 (0.7 - 0.98)	0.78 (0.6 - 0.91)	0.94 (0.7 - 1.0)
Sodium (mmoL/dL)	140 ± 3	138 ± 2	139 ± 2	139 ± 2
Hemoglobin (g/dL)	13.9 ± 0.9	14.2 ± 1.3	13.4 ± 0.7	14.2 ± 1.0
Total Cholesterol (mg/dL)	193 ± 38	202 ± 40	194 ± 42	200 ± 45
Reactive C protein (mg/dL)	1.15 (0.63 - 4.02)	1.71 (0.35 - 6.54)	1.24 (0.51 - 4.74)	1.09 (0.73 - 3.62)
NT-ProBNP (pg/mL)	686 (66 - 816)	60.5 (34 - 108)	96.0 (73 - 181)	839.5** (189 - 2271)
Troponin I (ng/mL)	0.684 (0.012 - 0.04)	0.012 (0.012 - 0.012)	0.012 (0.012 - 0.028)	0.038 (0.019 - 0.06)
LVEF (%)	54 ± 15	64 ± 3	64 ± 4	43 ± 10*
METS	9 ± 2.5	12 ± 3	9 ± 2	8 ± 2

NYHA: New York Heart Association; NT-ProBNP: N-terminal pro B-type natriuretic peptide; LVEF: left ventricular ejection fraction; METS: metabolic equivalent of task. Data expressed as mean ± standard deviation or percentage (%) for discrete variables and median and interquartile range for continuous variables with non-normal distribution.

* p < 0.001, Fisher's exact text.

** p < 0.001, Kruskal-Wallis one-way analysis of variance.

### The presence and extent of miocardial fibrose

**Figure 2:**
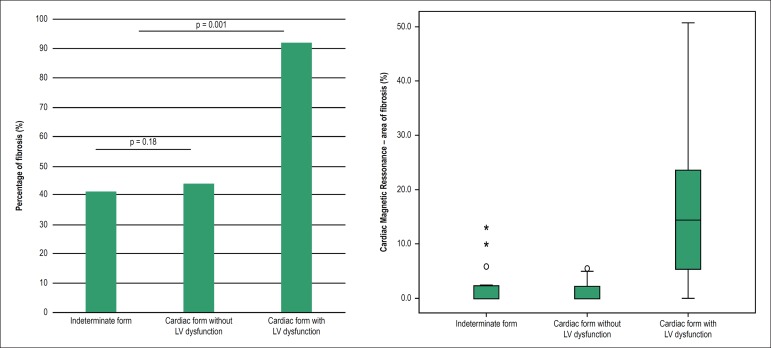
Myocardial fibrosis in the different clinical forms of Chagas’ disease. LV: left ventricular.

Late enhancement was found in 37 of the 58 subjects submitted to a CMR (64%). The percentage of the total area of ​​myocardium affected by fibrosis was 9.4% (IIQ: 2.4 - 18.4), and was located most frequently in the inferolateral and apical LV segments. The location of the fibrosis in the subnedocardial and transmural areas were the most prevalent (72%); however, transmural fibrosis occured more frequently in those with ventricular dysfunction; p = 0.001. Myocardial fibrosis was identified in 6 of the 17 individuals in the indeterminate form (41%; 95% CI: 23-66), which is a proportion similar to that observed in the cardiac form without LV dysfunction (44%, 7 of 16 individuals); p = 1.0. Among the individuals with fibrosis, the total area of the affected myocardium was 4.1% (IIQ: 2.1 - 10.7) in the indeterminate form versus 2.3% (IIQ: 1-5) in cardiac form without LV (p = 0.18). In those with ventricular dysfunction, the percentage of fibrosis was higher than in the other groups, occurring in 23 of the 25 subjects (92%), with a compromised area of 15.2% (IIQ: 7.8-25); p < 0.001 (Figure 2).

### The impact of myocardial fibrosis

**Figure 3:**
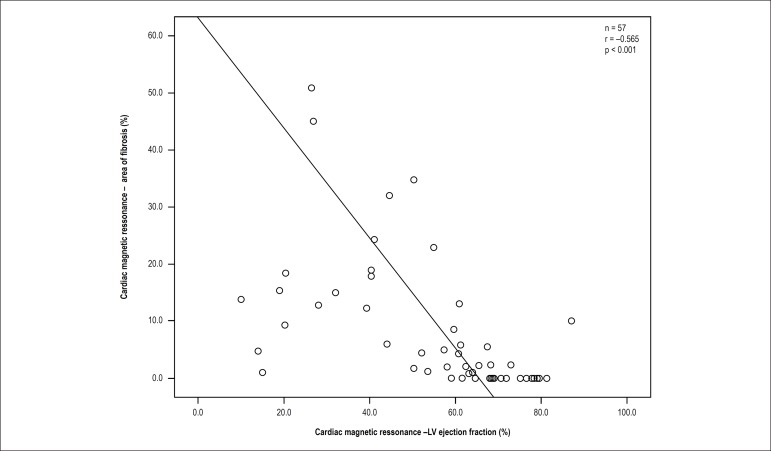
Linear regression analysis: influence of fibrosis on the left ventricular ejection fraction. LV: left ventricular.

The LV ejection fraction was significantly lower in individuals with late enhancement when compared to subjects without enhancement (69 ± 13 versus 48 ± 19%); p < 0.0001. A negative correlation was observed between the extent of fibrosis and ejection fraction (r = 0.565; p < 0.001). Through linear regression analysis, progressive reduction of the ejection fraction was observed at each percentage increase in the area affected by fibrosis. This analysis showed a regression coefficient (b) of -0.968, which corresponds to the estimated reduction in the ejection fraction of the LV at each 1% increase in the area of fibrosis (Figure 3).

There was a progressive increase in the amount of fibrosis in the different classes of the Rassi score, when subdivided into low, intermediate and high risk. The high-risk group had 13.8% (QI: 9 - 19) versus 4.9% (QI: 1 - 17) in the medium risk versus 0% (QI: 0 - 5) in the low risk group (p = 0.003). There was no difference in fibrotic mass between the low and intermediate risk classes (p = 0.19), nor was there a difference between intermediate and high risk (p = 1.0).

### Severity of the disease in its indeterminate form versus in its cardiac form without left ventricular dysfunction

The left ventricular ejection fraction (LVEF) in individuals in the indeterminate form was 72 ± 8%, which is similar to the LVEF of patients in the cardiac form without ventricular dysfunction 67 ± 6%; p = 0.09. There were no NT-proBNP levels (125 pg/ml versus 159 pg/ml, p = 0.61), ultrasensitive CRP (4.6 mg/L versus 2.5 mg/L; p = 0, 40), TNF-alpha (0.9 pg/ml versus 1.2 pg/ml, p = 0.56), interleukins (p = 0.35), IFN-gamma (2.7 pg/ml vs. 3 3 pg/ml; p = 0.56) and MET (10 vs. 9.4, p = 0.66) achieved through exercise testing and the location of late enhancement (p = 0.44) when comparing patients in the indeterminate form with those in the cardiac form without dysfunction (Table 2).

**Table 2:** Characteristics of the indeterminate form versus the cardiac form without left ventricular dysfunction

Variables	Indeterminate form (n = 17)	Cardiac form without ventricular dysfunction (n = 16)	p value
Rassi Score	2 (0 - 2)	6 (1 - 8)	0.30‡
Area of fibrosis from the CMR (%)	4.1 (2.1 - 10.7)	2.3 (1 - 5)	0.18‡
LV ejection fraction (%)	72 ± 8	67 ± 6	0.09†
Ventricular Tachycardia (%)	-	20	0.001*
METS	10 ± 3	9.4 ± 2	0.60†
Maximum VO_2_	35 ± 10	33 ± 7	0.47†
NT-ProBNP (pg/mL)	125 (34 - 108)	171 (73 - 181)	0.61‡
Ultrasensitive PCR (mg/L)	1.7 (0.35 - 6.5)	1.2 (0.51 - 4.7)	0.40‡
Troponin I (ng/mL)	0.012 (0.0 - 0.012)	0.012 (0.012 - 0.028)	0.31‡
IL-2 (pg/mL)	0.21 (0.03 - 0.55)	0.27 (0.03 - 0.96)	0.14‡
IL-4 (pg/mL)	0.62 (0.00 - 1.6)	0.37 (0.2 - 2.2)	0.83‡
IL-6 (pg/mL)	2.26 (1.39 - 4.35)	3.98 (2.01 - 6.22)	0.50‡
IL-10 (pg/mL)	0.44 (0.19 - 1.06)	0.63 (0.50 - 1.61)	1.47‡
TNF-alfa (pg/mL)	0.48 (0.14 - 1.15)	0.72 (0.58 - 2.71)	1.16‡
IFN-gama (pg/mL)	2.07 (1.30 - 4.35)	2.15 (1.69 - 6.73)	0.51‡

CMR: cardiac magnetic resonance; LV: left ventricular; METS: metabolic equivalent of task; Maximum VO_2_: Maximum Oxygen volume; NT-ProBNP: N-terminal pro B-type natriuretic peptide.

* Fisher's exact test;

† Student's t test;

‡ Mann-Whitney's test. Data expressed as mean ± standard deviation or percentage (%) for discrete variables and median and interquartile range for continuous variables with non-normal distribution.

All 17 individuals in the indeterminate form were classified as low risk according to the Rassi score. While 16 individuals were shown to be in the cardiac form without dysfunction, 12 (75%) were considered low risk and 4 were considered intermediate risk; p = 0.04.

## Discussion

The present study highlighted the presence of myocardial fibrosis in patients in the indeterminate form of Chagas’ disease. It was present in a frequency and extension similar to that of the group who had the disease in the cardiac form without ventricular dysfunction. Additionally, it was shown that ventricular function and clinical parameters are similar between these two forms.

CMR has been used for decades for the anatomical and functional evaluation of the heart. It is important due to the fact that it is non-invasive, does not use ionizing radiation, and has a high resolution, which allows for multiple studies concerning cardiac anatomy, function and tissue characterization with the late enhancement technique.^19-21^

Previous studies have validated the quantification of myocardial fibrosis using CMR in populations with Chagas' disease.^22,23^ In 2005, Rochitte et al.^17^ evaluated CMR with the use of the late enhancement technique in 51 patients with Chagas' heart disease, and found fibrosis in 68.6% of all of the individuals evaluated, and in 100% in those with ventricular tachycardia.^17^ Regueiro et al.^23^ found fibrosis in patients living outside the endemic area of the disease in a distribution of 7.4% of those in the indeterminate form, 15.8% of those in the cardiac form without ventricular dysfunction, and 52.4% in those with ventricular dysfunction.^23^ We found a percentage of fibrosis involvement similar to the previous study (64%), also showing progressive involvement in patients with LV dysfunction (92%).^17^ However, in addition to previous research, our data demonstrate a prevalence of enhancement as well as the percentage of fibrosis-related area, which is similar between the indeterminate form and the non-dysfunctional form of the LV. We found no difference in the subendocardial and transmural location of fibrosis.

The description of the cardiac ultrastructural changes that occur in the indeterminate phase of Chagas' disease were initially reported in individuals with positive serology during necropsy after accidental death.^12^ However, its designation as an indeterminate form was impaired since it was impossible to analyze the electrocardiographic alterations. In 1997, Andrade et al.,^24^ using a canine model, interpreted that the indeterminate form of the disease is characterized by a self-limited cycle of focal inflammatory alterations, with modulation and suppression of immune responses mediated by cells. Thus, they considered that the indeterminate form of Chagas' disease is characterized by a host-parasite equilibrium instead of a progressive damaging process.^24^ As early as 1978, Andrade et al.^13^ reported that chronic chagasic myocarditis lesions are not randomly distributed through the atrioventricular conduction system, but rather that there is a clear distribution of lesions in the conduction system.^13^ We now know that a large percentage of patients in the indeterminate form show evidence of cardiac involvement in the detailed, non-invasive evaluation.^25^ The data from the present study demonstrate that a CMR is not capable of differentiating the indeterminate form from the clinical form without LV dysfunction, since the percentage of fibrosis is similar between the two clinical forms. In this study, the percentage of fibrosis involvement in the indeterminate form (41.2%) was similar to the percentage in the cardiac form without LV dysfunction (43.8%).

Some limitations of the study should be recognized. No anatomical tests were performed to definitively rule out ischemic etiology as a cause of myocardial fibrosis. In order to minimize this possibility, an ergometric test was performed with all of the individuals, in addition to including the exclusion criteria of the presence of risk factors for atherosclerosis. Although it is recognized that to rule out coronary artery disease definitively, a coronary angiography would be necessary, the negative predictive value of the exercise test in these circumstances is very high. Coronary artery disease was excluded without performing a coronary angiography in order to avoid radiation and complications resulting from the procedure.

## Conclusion

The presence of fibrosis in the indeterminate form of Chagas' disease has a frequency and extension similar to that of the cardiac form without dysfunction, suggesting that the former is part of a subclinical disease spectrum, rather than lacking cardiac involvement. Thus, indeterminate and cardiac forms without dysfunction resemble each other and differ significantly from cardiac form with dysfunction.
